# Cost-effectiveness of Same-day Discharge Surgery for Primary Total Hip Arthroplasty: A Pragmatic Randomized Controlled Study

**DOI:** 10.3389/fpubh.2022.825727

**Published:** 2022-04-25

**Authors:** Yangyang Shi, Peipei Zhu, Jie Jia, Zengwu Shao, Shuhua Yang, Wei Chen, Ke Zhang, Wei Tong, Hongtao Tian

**Affiliations:** ^1^Department of Orthopaedics, Union Hospital, Tongji Medical College, Huazhong University of Science and Technology, Wuhan, China; ^2^The Third Hospital of Hebei Medical University, Shijiazhuang, China; ^3^Biostatistician at Causality Clinical Data Technology Co., Ltd., Wuhan, China

**Keywords:** total hip arthroplasty, same-day discharge surgery, efficacy, cost-effectiveness, quality of life

## Abstract

**Background:**

Total hip arthroplasty (THA) causes a great medical burden globally, and the same-day discharge (SDD) method has previously been considered to be cost saving. However, a standard cost-effectiveness analysis (CEA) in a randomized controlled trial (RCT) is needed to evaluated the benefits of SDD when performing THA from the perspective of both economic and clinical outcomes.

**Methods:**

Eighty-four participants undergoing primary THA were randomized to either the SDD group or the inpatient group. Outcomes were assessed by an independent orthopedist who was not in the surgical team, using the Oxford Hip Score (OHS), EuroQol 5D (EQ-5D), SF-36 scores and the quality-adjusted life years (QALYs). All the cost information was also collected.

**Results:**

The mean stay of patients in the SDD group was 21.70 ± 3.45 h, while the inpatient group was 78.15 ± 26.36 h. This trial did not detect any significant differences in OHS and QALYs. The total cost in the SDD group was significantly lower than that in the inpatient group (¥69,771.27 ± 6,608.00 vs. ¥80,666.17 ± 8,421.96, *p* < 0.001). From the perspective of total cost, when measuring OHS, the incremental effect was −0.12 and the incremental cost was –¥10,894.90. The mean incremental cost-effectiveness ratio (ICER) was 90,790.83. When measuring QALYs, the incremental effect was 0.02, and the ICER was negative. Sensitivity analysis produced similar results.

**Conclusions:**

SDD has an acceptable likelihood of being more cost-effective than the traditional inpatient option. After conducting cost–utility analysis, SDD resulted in better QALYs, while significantly reducing the total cost.

## Introduction

Total hip arthroplasty (THA) is the first-choice treatment for many hip joint diseases. There were 378,089 THAs in the US in 2015 (1), with a cost of over 22,000 US dollars per procedure (2). In China, there were around 900,000 THAs in 2019. Thus, THAs result in substantial medical costs and are placing pressure on national budgets in both developed and developing countries. To reduce the cost, US Medicare launched the mandatory Comprehensive Care for Joint Replacement bundled payment model, which resulted in substantial hospital savings and reduced Medicare payments (3, 4). China adopted some strategies such as the diagnosis-related group (DRG) payment system to reduce the cost (5). Moreover, the economic environment greatly influences the number of joint arthroplasty procedures (1) and extreme poverty rises for the first time since 1998 due to the spread of COVID-19. In short, it is vital to reduce the medical cost both for the government and for individuals under these conditions.

Length of stay (LOS) in hospital is a crucial determinant of medical cost, and minimizing LOS could result in significant cost savings for arthroplasty (6). In the past 10 years, joint replacement has been performed on strictly selected patients on a same-day discharge (SDD) basis in the US and Europe, and studies showed similar or even better outcomes compared to inpatient operation, resulting in cost savings of up to 30% per case (7, 8). Based on this, the US government removed arthroplasty from the inpatient operation list in Jan. 2018, in an attempt to move toward outpatient surgeries. However, data or polices from a high-income country cannot be extrapolated to the whole world, especially low-income countries. Moreover, studies have shown conflicting results regarding surgery effects, complications and adverse events when comparing between classical inpatient surgery and SDD for arthroplasty (9–11). Therefore, a standard and comprehensive cost-effectiveness study is needed, to help surgeons decide between outpatient or inpatient THA, balancing outcomes and costs (12). Recently, a computer-based retrospective study revealed that outpatient THA was cheaper but less effective in terms of total utility, and more cost-effective than inpatient THA within a specific willing-to-pay (WTP) threshold (13). However, to the best of our knowledge, no randomized controlled trial has been performed to analyze the cost-effectiveness of primary THA on a SDD basis. In addition, no systematic evaluation of SDD THA has ever been reported in China.

In this study, we evaluated the cost-effectiveness of SDD compared with that of regular care for patients who needed primary THA by analyzing the effect using the Oxford hip score (OHS), medical costs (both out-of-pocket and reimbursed), mean incremental cost-effectiveness ratio (ICER), and quality-adjusted life years (QALYs) at 6-month follow up. By a standard cost-effectiveness analysis (CEA), we hope to help physicians and government to look at SDD in a more accurate way.

## Materials and Methods

### Study Design and Participants

A prospective RCT was conducted between Oct 2017 and Aug 2019. Prior to the start of this study, the study protocol was approved by Wuhan Union Hospital Ethical Committee (0086-01). All participants provided written informed consent. Patients qualified for inclusion in the study if they met the following criteria: undergoing unilateral primary THA; having the ability to understand the relevant treatment process; aged between 18 and 75 years; a body mass index (BMI) ≤ 40 kg/m^2^; hemoglobin ≥ 12 g/dL; American Society of Anesthesiologists (ASA) physical status classification of I or II; and no ongoing infection or blood coagulation disorders. Those with a history of coronary artery disease, chronic obstructive pulmonary disease, arrhythmias, or untreated obstructive sleep apnea were excluded. Eligible individuals were randomly assigned (1:1) to an inpatient THA group or an SDD THA group. SDD-THA was defined as admission, surgery, and discharge within 24 h, whereas the inpatients stayed in hospital for more than 1 day.

### Treatment

Preoperatively, patients undergoing SDD-THA received information in the form of a teaching class conducted by a bedside clinician, which included the protocol, matters needing attention, exercise training, and home-based rehabilitation. All operations were performed by the same surgical team through a posterolateral approach. Celebrex 400 mg orally was used as routine analgesia before surgery. Cefazolin (1.0 g) and Tranexamic Acid (0.4 g) were administered 30 min prior to skin incision. A uniform perioperative multimodal pain management protocol was established by cocktail periarticular injection before wound closure, which consisted of Flurbiprofen axetil (50 mg) and Ropivacaine (200 mg). To avoid venous thromboembolism (VTE), all participants were encouraged to perform ankle pumping and quadriceps-setting exercises immediately. To relieve the pain, a multimodal postoperative pain management protocol was used, with all patients being given 200 mg Celebrex orally every 12 h and 5 mg/325 mg Hydrocodone/acetaminophen orally every 6 h. Patients undergoing SDD-THA received an additional IV dosage of antibiotics before discharge and 4 doses of 750 mg Cefaclor orally every 12 h after discharge.

Regardless of group assignment, all participants met the same criteria before discharge: general wellbeing, a dry wound, capable of independent transfers, and able to walk one hundred feet. On the first day after discharge, a visiting nurse called each patient to confirm they were doing well. All patients were followed up for 6 months, whether it was by phone, WeChat, or other means.

### Outcomes

The primary outcome was the OHS with 12 questions reflecting the different aspects of hip function. Each question was scored from 0 to 4, with 4 representing the best outcome or least symptoms (14). The secondary outcome was QALYs which was calculated as the outcome in cost utility analysis, as proposed by the CHEERS and CEA guidelines. QALYs is a parameter used to evaluate quality of life (QOL) and life year gain. The values of QOL were estimated by EQ-5D (15). The score for the EQ-5D ranges from −0.59 to 1.00, with a higher score indicating a better quality of life. QALYs health benefits were estimated by calculating the area under the curve (AUC) of the EQ-5D for linear interpolation over the whole 6-month period, and was calculated as: QALYs = EQ-5D × 0.5.

### Total Costs

In cost-effectiveness analysis, the total costs includes direct costs and indirect costs. Direct costs are all costs that are attributable to health care intervention, which can be divided into direct medical costs and direct non-medical costs. Direct medical costs are all treatment costs incurred during hospitalization, while direct non-medical costs are payments other those for the medical institute, e.g., transportation costs and nutritional costs. Indirect costs represent economic losses resulting from hospitalization due to disease, e.g., the loss of working opportunity. The subjects of this study were hospitalized patients, whose costs were mainly reflected in direct medical costs, while direct non-medical costs and indirect costs were negligible, which had little impact on the research results. In addition, the direct non-medical costs and indirect costs vary among individuals and gathering this information by questionnaire was usually challenging in real world scenario. Therefore, the total costs of this study is the direct medical costs, including operating room (OR) supply costs, surgical facility costs, hospital room costs, examination costs, laboratory costs, medication costs, and therapy costs. The total costs also could be calculated by summing up reimbursed costs and out of pocket costs. Reimbursed costs refer to the cost reimbursed by the national medical insurance and commercial insurance, and out of pocket costs refer to the costs that patients need to pay by themselves after reimbursement.

### Out-of-Pocket Costs

The out-of-pocket and reimbursed charges added up to the total costs. From the patients' standpoint, the out-of-pocket costs meant the final total costs, which were considered to be the patients' primary concern.

### Statistical Analysis

The study did not have enough power to statistically test for differences in health economy outcomes. As a result, we adopted a probabilistic approach to healthy economic inference, with the aim of informing decision makers about probability rather than statistical significance. This study was also an exploratory study, based on this fact, we did not set any hypothesis and the sample size would be pre-determined by clinical experience. All analyses were conducted according to the intention-to-treat (ITT) principle. ITT consists of keeping all patients included, in their initial group in the case of randomization, to perform final analysis of a study. ITT approach was adopted in this trial in which patients were analyzed as randomized regardless of the treatment actually received.

In the CEA, the resulting estimates were incremental cost-effectiveness or cost-utility ratios (ICER/ICUR). Incremental cost over 6 months was divided by incremental effect (treatment response or QALYs, respectively): ICER/ICUR = (Cost_SDD_ - Cost_inpatient_)/(Effects_SDD_ - Effect_inpatient_). Effects were calculated separately utilizing OHS and QALYs, and the ICER/ICUR was based on the incremental costs per unit effect (OHS and QALYs) gained.

The sampling uncertainty of the ICER/ICUR estimate was calculated by using a non-parametric bootstrapping method in each of the 5,000 iterations. These estimates were visualized using the cost-effectiveness plane and the cost-effectiveness acceptability curve. The cost-effectiveness plane showed four quadrants of the uncertainty of cost and effect, namely the southeast (intervention costs lower than the control group, and the effect is better), northeast (intervention costs higher than control group, but effect is better), southwest (intervention costs lower than the control group, but the effect is worse) and northwest (intervention costs higher than the control group, but the effect is worse). Cost-effectiveness acceptability curves were plotted to show the likelihood that interventions would be cost-effective according to different WTP thresholds. All statistical analyses were performing using R software (Version 3.6.1; 2019 The R Foundation for Statistical Computing).

### Sensitivity Analysis

A sensitivity analysis was conducted to evaluate the robustness of the findings. Sometimes, the EQ-5D could be replaced by the SF-6D in measuring QALYs. Some studies used these two items together when estimating QALYs of patients after joint replacements (16). Since the direct use of the SF-6D questionnaire is not recommended, we used the Japanese SF-36 (version 2) to calculate QALYs, which are reported to be more sensitive to QALY variations when treating patients with osteoarthritis in China (17).

## Results

### Patients Characteristics

**Figure 1:**
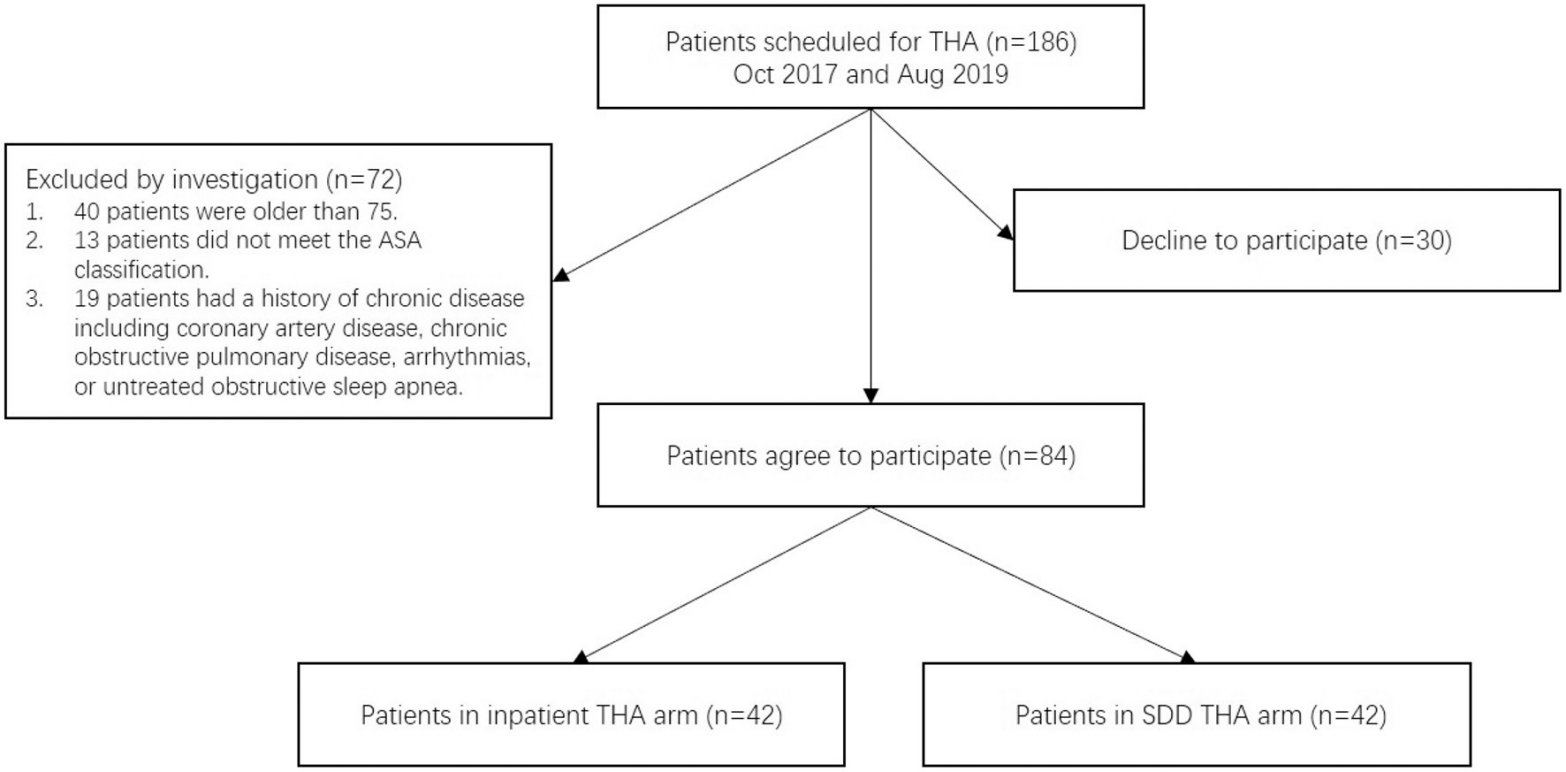
Flow chart of participants enrolled in the study with details on loss of data.

Eighty-four participants were recruited from 186 primary THAs at our institution. The remaining 102 patients did not meet the eligibility criteria or decline to participate (Figure 1). Four patients of the SDD group failed to be discharged within the first day, giving a failure rate of 9.52%. The failures did not affect the results of the descriptive statistics according to any socio-demographic factor or any health status factor which was reported at the baseline. Based on the intent-to-treat principle, we still took them into account. All the participants completed a 6-month follow-up, and 84 (100%) of them completed the questionnaires. The characteristics of 84 participants are shown in Table 1.

**Table 1:** Characteristics of the THA patients in the two groups at baseline.

**Variable**	**Inpatient (*n* = 42)**	**SDD (*n* = 42)**	***P*-value**
Age (years)	54.50 ± 11.52	53.81 ± 12.21	0.79
**Sex**			0.83
Female	23 (54.76%)	22 (52.38%)	
Male	19 (45.24%)	20 (47.62%)	
BMI (kg/m^2^)	27.12 ± 3.67	26.78 ± 3.37	0.66
ASA	1.48 ± 0.51	1.52 ± 0.51	0.67
**Comorbidity**			
Hypertension	11	9	0.61
Diabetes	8	9	0.79
Coronary heart disease	6	7	0.76
LOS (hours)	78.15 ± 26.36	21.70 ± 3.45	<0.001
OHS	21.14 ± 2.92	21.05 ± 3.02	0.88
EQ-5D	0.29 ± 0.11	0.29 ± 0.09	0.91

*BMI, body mass index; ASA, American Society of Anesthesiologists classification; OHS, Oxford hip score (numerical rating scale); LOS, length of stay*.

### Effects

The mean OHS at the 6-month follow-up was 38.98 ± 3.06 in the SDD group (95%CI 38.02–39.93), while in the inpatient group it was 39.10 ± 2.64 (95%CI 38.27–39.82). Between groups, there was no statistically-significant difference (*p* = 0.85). The mean QALY at the 6-month follow-up was 0.34 ± 0.08 in the SDD group and 0.32 ± 0.07 in the inpatient group (95%CI 0.32–0.37 and 0.30–0.34, respectively), again with no statistically-significant difference (*p* = 0.19). The incremental effects of OHS and QALY were −0.12 and 0.02.

### Costs

**Figure 2:**
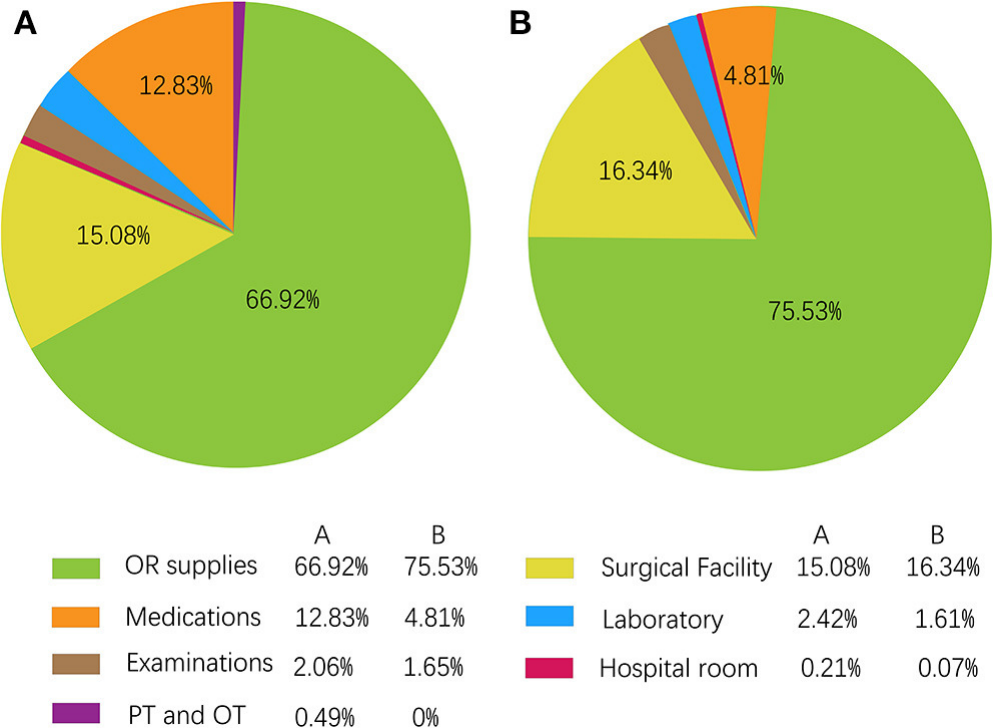
The composition of each cost as a proportion of total charge. **(A)** Inpatient group; **(B)** SDD group.

At baseline, the mean total cost was ¥69,771.27 ± 6,608.00 in the SDD group and ¥80,666.17 ± 8,421.96 in the inpatient group, and the incremental cost was −10,894.90, indicating that the total cost for the SDD group was lower than for the inpatient group and the result was statistically significant (*p* < 0.001). Table 2 lists all the cost details and compares them by categories. In other categories of charge, apart from OR supplies and the surgical facility fee, all the others indicated the same result as the total cost. This can probably be explained by the shorter stay in hospital resulting in a lower cost. Figure 2 more intuitively demonstrates the differences in total treatment costs between the two groups as well as the differences in costs between each category.

**Table 2:** Mean costs over the 6-month follow-up between the SDD group and the inpatient group (based on intent-to-treat population, *n* = 84).

	**Inpatient (*n* = 42) Mean ±SD, ¥**	**SDD (*n* = 42 Mean ±SD, ¥**	**Incremental cost difference, ¥**	***P*-value**
Total charges	80,666.17 ± 8,421.96	69,771.27 ± 6,608.00	−10,894.90	<0.001
Reimbursed	55,010.42 ± 13,042.44	48,554.52 ± 9,264.96	−6,455.90	0.01
Out-of-pocket	25,655.75 ± 11,908.91	21,216.75 ± 7,820.36	−4,439.00	0.05
OR supplies^a^	53,981.56 ± 7,714.41	52,646.64 ± 6,832.78	−1,334.92	0.40
Surgical facility fee^b^	12,161.78 ± 2,299.03	11,431.36 ± 1,444.84	−730.42	0.09
Hospital room	167.86 ± 55.07	51.48 ± 13.96	−1,116.38	<0.001
Examinations^c^	1,659.37 ± 743.21	1,144.95 ± 336.97	−514.42	<0.001
Laboratory charges	1,951.53 ± 474.25	1,131.24 ± 261.80	−820.29	<0.001
Medications	10,345.40 ± 2430.09	3,365.61 ± 1,134.37	−6,979.79	<0.001
PT and OT	398.67 ± 158.28	0	−369.94	<0.001

*OR, operating room; OT, occupational therapy; PT, physical therapy*.

a *OR supplies includes all the medical materials utilized during the operation process*.

b *Surgical facility fee includes anesthesia, OR utilized and recovery room fees*.

c *Examinations includes electrocardiography, ultrasonic cardiogram, X-ray, Computed Tomography (CT), etc*.

### Cost-effectiveness and Cost-utility

**Figure 3:**
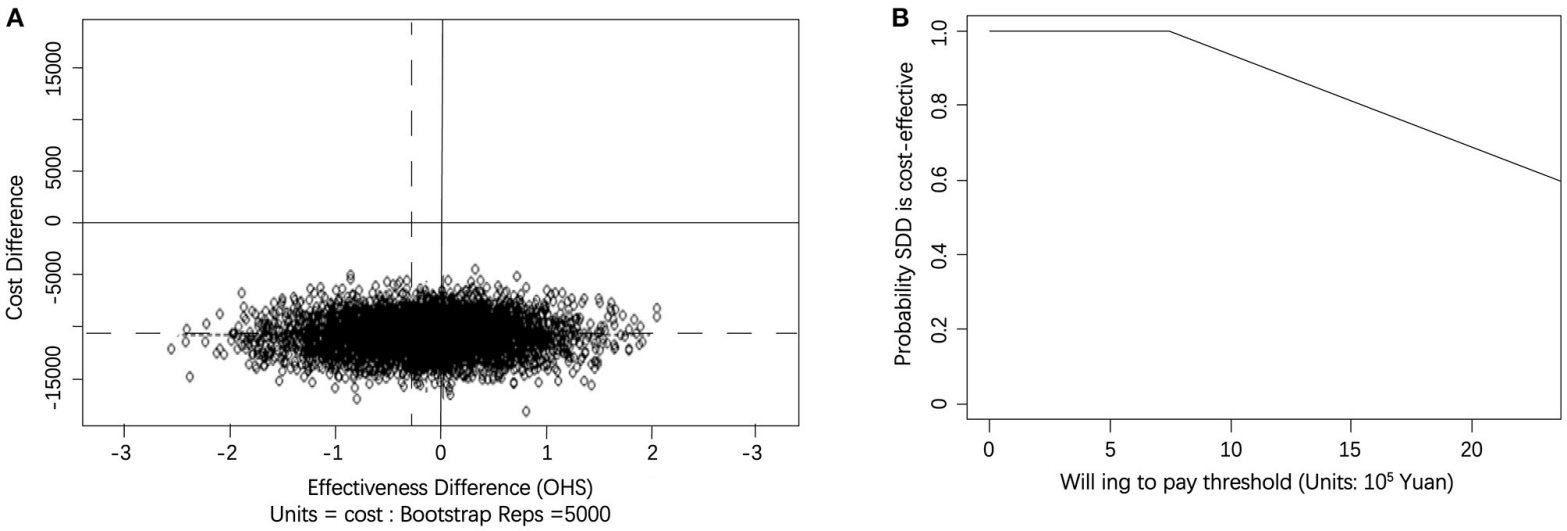
**(A)** Scatterplot of 5,000 replicates of the ICER (mean differences in total cost in OHS) on the cost-effectiveness plane. The circles in northwest quadrants represent trials in which SDD-THA costs lower than the inpatient THA, but the effect is worse. The circles in northwest quadrants represent that SDD-THA was less costly and more effective than the inpatient THA. **(B)** Cost-effectiveness acceptability curve showing the probability of the SDD procedure being cost-effective at varying WTP ceilings (based on 5,000 replicates of the ICER using mean differences in total cost).

Table 3 shows the incremental cost, incremental effect and mean ICER based on a calculation of 5,000 bootstrap simulations for both cost–utility analysis and sensitivity analysis. According to OHS, the SDD group achieved a lower mean total cost (–¥10,899.38) compared with the inpatient group, while it resulted in a decline in mean effect (−0.14). In the related cost-effectiveness plane, after processing 5,000 bootstrap simulations, as shown in Figure 3A, almost half of the bootstrapped ICERs were mapped in the south west quadrant which indicated that SDD saved money, but may reduce the treatment effect. Meanwhile, the remaining half of the bootstrapped ICERs fell into the south east quadrant, which indicated that SDD not only saved money, but also obtained a better effect. Based on this calculation, an acceptability curve was generated as shown in Figure 3B. At a WTP ceiling value of ¥0, the probability that SDD being cost effective was 100%, and this trend ketp on when the WTP was around ¥800,000. However, when WTP went on increasing, the probability would decrease accordingly. For example, when WTP was increased to ¥2,000,000, the probability that SDD could be regarded as more cost-effective than inpatient treatment was reduced to approximately 65%.

**Table 3:** Differences in treatment response and QALYs outcomes between inpatient and SDD groups at the 6-month follow-up (based on 5,000 bootstrap simulations).

	**Incremental cost, ¥**	**Incremental effect**	**Mean ICER**	**Distribution over the ICER plane**			
	**(95%CI)**	**(95%CI)**		**NE**	**NW**	**SE**	**SW**
Cost-effectiveness OHS at 6 months	−10,899.38 (−14,183.34, −7,342.22)	−0.14 (−1.49, −1.08)	77,852.71			45%	55%
Cost-utility, EQ-5D, QALYs	−10,899.38 (−14,183.34, −7,342.22)	0.02 (−0.05, 0.11)	Dominate			85%	15%
Sensitivity analysis SF-6D, QALYs	−10,899.38 (−14,183.34, −7,342.22)	0.03 (0.01, 0.05)	Dominate			90%	10%

*NE, northeast; NW, northwest; SE, southeast; SW, southwest*.

**Figure 4:**
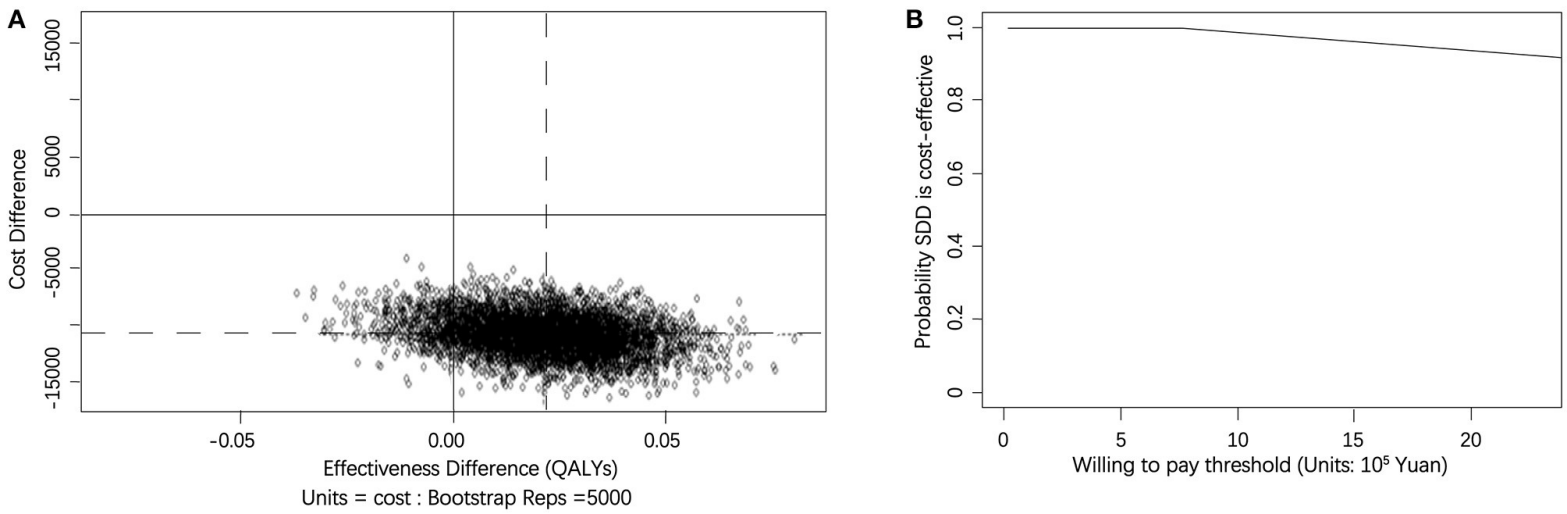
**(A)** Scatterplot of 5,000 replicates of the ICER (mean differences in total cost in QALYs) on the cost-effectiveness plane. The circles in northwest quadrants represent trials in which SDD-THA costs lower than the inpatient THA, but the effect is worse. The circles in northwest quadrants represent that SDD-THA was less costly and more effective than the inpatient THA. **(B)** Cost-effectiveness acceptability curve showing the probability of the SDD procedure being cost-effective at varying WTP ceilings (based on 5,000 replicates of the ICER using mean differences in total cost and QALYs).

When calculating the mean ICER of cost–utility using QALYs, we obtained a negative result. The cost-effectiveness plane revealed the ICER distribution based on QALYs, as shown in Figure 4A. Most of the ICERs were mapped to the south east quadrant, which indicated that SDD produced a better outcome and lower cost. Figure 4B also showed the acceptability curve based on calculation of QALYs. As WTP was between ¥ 0 and 800,000, the probability of SDD being cost-effective remained extremely closed to 100%. Similar to Figure 3B, when WTP went on increasing, the probability of SDD being cost-effective would decrease accordingly as well.

### Sensitivity Analyses

Using the SF-6D as an alternative measurement of QALYs produced a similar result as using EQ-5D. At the 6-month follow up, the incremental effect was 0.03 (95%: 0.01–0.05). When calculating the mean ICER, we obtained a negative result which meant that the SDD group dominated the inpatient group. Up to 90% of the ICERs were mapping in the south east quadrant. We also obtained another acceptability curve based on calculation of QALYs using SF-6D which was similar to Figure 4B. This indicated that as WTP increased, the probability of SDD being cost-effective remained extremely close to 100% and the results showed no differences with our findings using EQ-5D.

## Discussion

Our study was designed to estimate the cost-effectiveness and cost utility of same-day THA compared with traditional THA surgery. The results showed that SDD exhibited similar effects and lower cost compared with the traditional inpatient group. In addition, cost-effective analysis demonstrated that effectiveness varied at different WTP thresholds. To the best of our knowledge, this is the first CEA analysis on same-day THA, and the first same-day THA study carried out in China.

SDD has been performed for almost 20 years in the US and Europe, and a systematic review which included 1,009 patients undergoing SDD found no significant difference between inpatient and SDD groups regarding readmissions and complications (18). However, age has some influence on the outcome of the two operations. Berger et al. included patients aged from 50 to 80 years, and found more complications in the SDD group than the inpatient group (10), while another study which only included patients aged between 42 and 64 years (mean age 55) found no significant difference regarding the outcome or complications (19), which is in line with our data (mean age 53 years). This is possibly because health-related quality of life measured by EQ-5D has a negative correlation with age (20). Therefore, SDD is probably more feasible for younger patients which is almost 10 years younger than the general age of THA patients (around 63 years), so patients of varied age are needed to be included in further studies to draw definite conclusions.

SDD has been reported to reduce LOS and is a useful method to reduce the cost. Molloy et al. found that shortening the LOS could lead to a 17.6% decrease in the cost of THA by reducing the LOS from 4.06 to 2.97 days (2). Bertin et al. found that charges of SDD group were $2,465 less (*p* = 0.02) than that of inpatient THAs, demonstrating a 10.68% drop (21), while other studies showed a 30–50% decrement, which may be due to the 71.27% reduction of surgical facility fees (7, 22). In our case, we reduced the LOS from 78.15 h for inpatients to 21.70 h in SDD patients, and found 13.51% reduced expenditure (¥10,894.90) in the SDD group. Moreover, the most obvious reduction was the medication fee (4.82 vs. 12.82%) in our study, while there were no differences in OR supplies, which is reasonable and consistent with previous studies.

In our study, we evaluated the out-of-pocket and reimbursement portions separately. First, we showed that out-of-pocket expenses were decreased by 4,439.00 RMB, accounting for 7% of the 2019 GDP per capita in China. Interestingly, we found that the reimbursement portion decreased more significantly in the SDD group compared with the out-of-pocket portion (6,455.90 RMB vs. 4,439.00 RMB). This may be because the medication fee dropped most significantly, which are mostly on the reimbursement list. Considering that 900,000 procedures of hip arthroplasty were performed in China in 2019 and a projection of 572,000 in the US in 2030 (23), day surgery could bring great savings for the government. Therefore, our study provides a rationale to perform THA on an SDD basis especially under the current circumstances when we are undergoing a COVID-19 outbreak and thus an economic crisis.

There is an ever-increasing impetus to reduce the medical burden for every government, regarding medical resources. In developing countries such as China, the medical resources are especially limited: the number of hospital beds in China is 28.49 ± 17.10/100,000 people (24), and this is far less than that of developed countries. SDD surgery maximizes bed utilization, which could make fuller use of the medical resources, and specifically benefit developing countries.

We also found that different WTP thresholds affected the ICER. Assuming a WTP of ¥2,000,000, the likelihood of SDD being cost-effective was approximately 65% for gaining one unit of OHS. If the likelihood of SDD being cost-effective is higher than 90%, a WTP of approximate ¥1,200,000 or lower for gaining one unit of OHS would make the SDD option cost-effective. If the WTP increased, the probability of SDD being cost-effective would decrease. Meanwhile the result of OHS gains were not mirrored in EQ-5D QALYs gains. We observed that the SDD group dominated the inpatient group when calculating cost–utility, which meant that the SDD option was not only cost-saving, but also presented better result in estimating patients' quality of life. The sensitivity analysis showed similar result.

This study has several limitations. First, we mainly compared the direct medical costs of the surgery, although direct non-medical costs and indirect costs were negligible, which had little impact on the research results. Second, the follow-up time was only 6 months, which is relatively short, and thus we have no data regarding any differences in long-term complications and revision rate between two groups. Third, this is a single center study in one country, and as health policies differ greatly between regions and countries around the world, the data would probably be different. Therefore, a multicenter long-term follow-up study is needed in the future.

## Conclusion

We found a reduced cost but similar surgical effects of THA, and no complications, when performed as SDD surgery compared to regular inpatient procedures in a short-term follow-up pragmatic RCT study. Moreover, the probability of this option being cost effective varied depending on the willing-to-pay threshold.

## Data Availability Statement

The original contributions presented in the study are included in the article/Supplementary Material, further inquiries can be directed to the corresponding author/s.

## Ethics Statement

The studies involving human participants were reviewed and approved by Wuhan Union Hospital. The patients/participants provided their written informed consent to participate in this study.

## Author Contributions

HT and WT was responsible for conception and design of the study. JJ contributed to data collection. YS and PZ drafted the manuscript. WC and KZ contributed to manuscript preparation and data analysis. ZS and SY contributed to revision of the manuscript. All authors contributed to either the conception, design, data collection, analysis, and read and approved the final manuscript.

## Funding

This study was supported by the National Natural Science Foundation of China (Nos. 82072509 and 81702157).

## Conflict of Interest

KZ was employed by Biostatistician at Causality Clinical Data Technology Co., Ltd. The remaining authors declare that the research was conducted in the absence of any commercial or financial relationships that could be construed as a potential conflict of interest.

## Publisher's Note

All claims expressed in this article are solely those of the authors and do not necessarily represent those of their affiliated organizations, or those of the publisher, the editors and the reviewers. Any product that may be evaluated in this article, or claim that may be made by its manufacturer, is not guaranteed or endorsed by the publisher.
